# NFATc3 plays an oncogenic role in oral/oropharyngeal squamous cell carcinomas by promoting cancer stemness via expression of OCT4

**DOI:** 10.18632/oncotarget.26774

**Published:** 2019-03-19

**Authors:** Sung Hee Lee, Calvin Kieu, Charlotte Ellen Martin, Jiho Han, Wei Chen, Jin Seok Kim, Mo K. Kang, Reuben H. Kim, No-Hee Park, Yong Kim, Ki-Hyuk Shin

**Affiliations:** ^1^ The Shapiro Family Laboratory of Viral Oncology and Aging Research, UCLA School of Dentistry, Los Angeles 90095, CA, USA; ^2^ Laboratory of Stem Cell and Cancer Epigenetics, UCLA School of Dentistry, Los Angeles 90095, CA, USA; ^3^ UCLA Jonsson Comprehensive Cancer Center, Los Angeles 90095, CA, USA; ^4^ Department of Medicine, David Geffen School of Medicine at UCLA, Los Angeles 90095, CA, USA; ^5^ UCLA Broad Stem Cell Research Center, Box 957357, Los Angeles 90095, CA, USA

**Keywords:** NFATc3, OSCC, cancer stem cells, OCT4

## Abstract

Nuclear factor of activated T cells (NFATc1-c4), a family of transcription factors, is involved in many biological processes by regulating various downstream target genes. However, their role in cancer progression remains controversial. We here report that NFATc3 is the dominant isoform of NFAT in human oral epithelial cells, and its expression was increased in a stepwise manner during the progression of oral/oropharyngeal squamous cell carcinoma (OSCC). More importantly, NFATc3 was highly enriched in self-renewing cancer stem-like cells (CSCs) of OSCC. Increased expression of NFATc3 was required for the maintenance of CSC self-renewal, as NFATc3 inhibition suppressed tumor sphere formation in OSCC cells. Conversely, ectopic NFATc3 expression in non-tumorigenic immortalized oral epithelial cells resulted in the acquisition of self-renewal and increase in CSC phenotype, such as enhanced ALDH1^HIGH^ cell population, mobility and drug resistance, indicating the functional role of NFATc3 in the maintenance of CSC phenotype. NFATc3 expression also converted the non-tumorigenic oral epithelial cells to malignant phenotypes. Mechanistic investigations further reveal that NFATc3 binds to the promoter of *OCT4*, a stemness transcription factor, for its activation, thereby promoting CSC phenotype. Moreover, suppression of *OCT4* abrogated CSC phenotype in the cell with ectopic NFATc3 overexpression and OSCC, and ectopic OCT4 expression sufficiently induced CSC phenotype. Our study indicates that NFATc3 plays an important role in the maintenance of cancer stemness and OSCC progression via novel NFATc3-OCT4 axis, suggesting that this axis may be a potential therapeutic target for OSCC CSCs.

## INTRODUCTION

OSCC, a common malignant tumor of the head and neck is the 6th most common cancer worldwide [1]. Projected 5-year survival for OSCC is approximately 50%. Recently, incidence of oral cancer among young adults has been alarmingly elevated [2, 3], indicating that OSCC is an emerging public health concern. Clinically well-defined lesions, such as leukoplakia that is histologically classified as dysplastic or non-dysplastic leukoplakia, often precede OSCC. Dysplastic leukoplakia is defined as oral premalignant lesion and associated with a likely progression to cancer; however, it is not an accurate predictor of cancer risk [4, 5]. Early-stage tumors can usually be managed through surgery and radiotherapy. However, successful treatment is inversely proportional to the extent of the disease at the time of treatment. A combination of chemotherapy and radiation therapy is very effective in treating early-stage tumor; however, its therapeutic effect on advanced tumors remains poorly [6], urgently demanding new directions in therapeutics.

Cancer stem cells (CSCs or also known as tumor-initiating cells) are small subpopulations of tumor cells that retain characteristics similar to normal stem cells, and their presence are reported in various primary tumors and established cancer cell lines, including OSCC [7, 8]. They play a crucial role in tumorigenicity, metastasis, and recurrence and thus are considered as the root of the cancer [9]. Properties and stemness of CSC can be maintained by several endogenous signaling pathways, such as Notch, Hedgehog, Wnt, Bmi1, Pten, Bmp, and TGF-β [10–16] which are frequently activated in human cancers [11, 17, 18]. In addition, we recently reported additional novel oral CSC molecular determinants, such as histone demethylases [19], microRNA [20], human papilloma viruses [20], and calcium (Ca^2+^) signalling [21]. Therefore, advancing our understanding of the molecular properties and signaling pathways unique to oral CSCs is crucial for developing a new generation of targeted and effective therapies for OSCC.

NFAT signaling plays an oncogenic role in tumorigenesis [22]. Four members of the NFAT family (*i.e*., NFATc1-c4) that are regulated by Ca^2+^ signaling have been identified [23]. They share a highly-conserved DNA binding domain, but depend on interacting partners including other transcription factors and co-activators for their target gene specificity. However, other studies also demonstrated tumor suppressive activity of NFAT, indicating their differential roles in different tumor types [24, 25]. Interestingly, recent studies have demonstrated the crucial role of NFAT in the maintenance of CSCs in various human cancers, such as colon, melanoma, pancreas and lung [26–29]. Thus, the roles of NFAT in tumorigenesis and cancer stemness remain obscure, especially in OSCC progression and stemness.

In the present study, we report for the first time that NFATc3 is the dominant isoform, and its expression is elevated in a stepwise manner in OSCC progression and further enriched in OSCC CSC populations. We further provided evidence that NFATc3 promotes CSC phenotype by upregulating OCT4, suggesting a novel CSC regulatory mechanism by NFATc3-OCT4 axis.

## RESULTS

### NFATc3, the dominant isoform of NFAT, is upregulated in OSCCs and further enriched in OSCC tumor spheres

**Figure 1:**
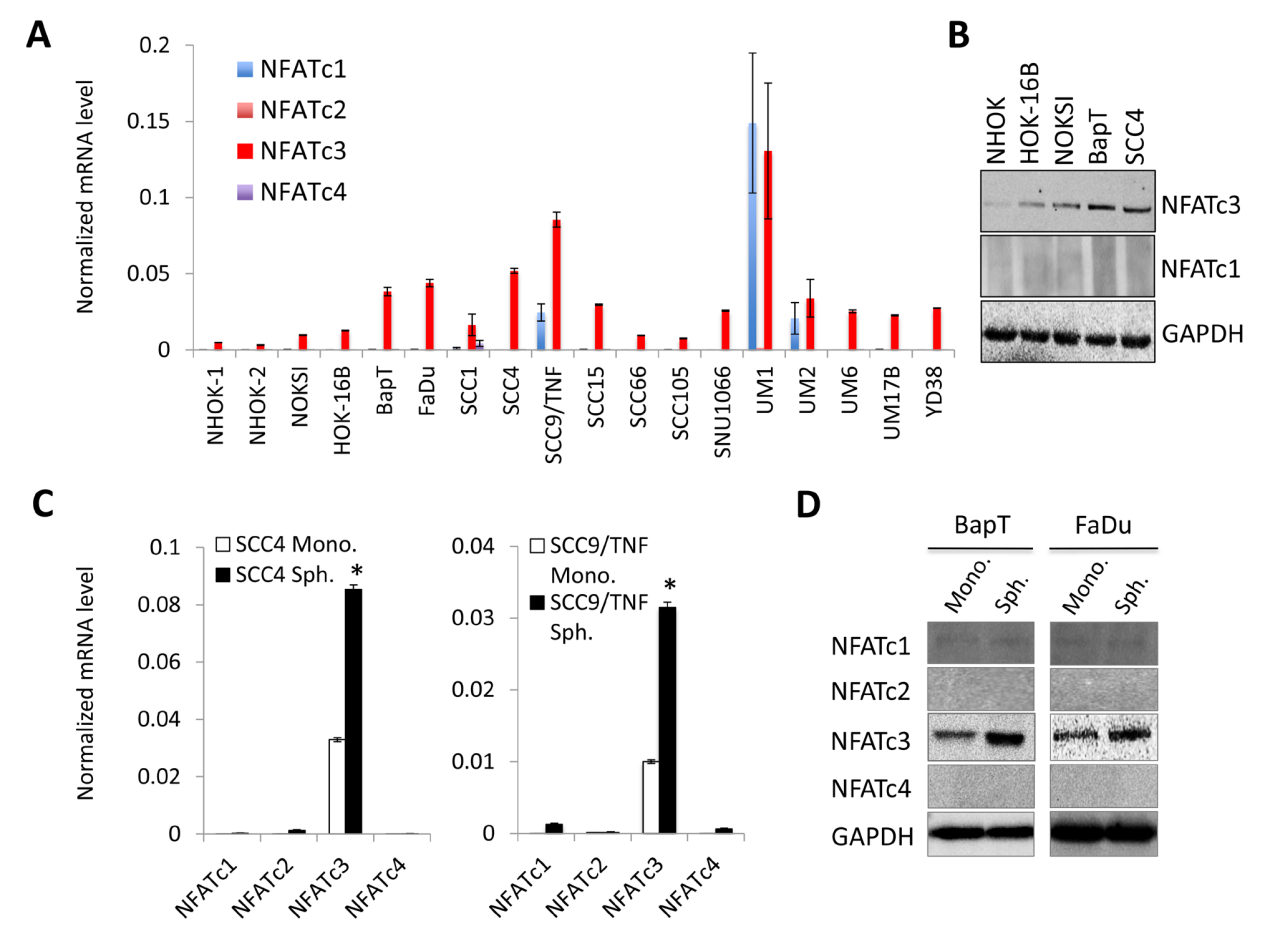
NFATc3 is increased in OSCC and further enriched in OSCC tumor spheres (**A**) Level of NFAT isoforms (NFATc1, NFATc2, NFATc3, and NFATc4) was determined in two strains of normal human oral keratinocyte (NHOK-1 and -2), 2 precancerous, non-tumorigenic immortalized oral epithelial cell lines (HOK-16B and NOKSI) and 10 OSCC cell lines (BapT, SCC1, SCC4, SCC9/TNF, SCC15, UM1, UM2, UM6, UM17B, and FaDu) by qPCR. Levels of NFAT isoforms were normalized to GAPDH. (**B**) Level of NFATc3 protein was assessed in normal (NHOK), precancerous (HOK-16B and NOKSI) and OSCC cells (BapT and SCC4) by Western blot analysis. GAPDH was used as loading control. (**C**) Expression of NFAT isoforms was assessed in tumor spheres (Sph.) and their corresponding adherent monolayer cells (Mono.) derived from multiple OSCC cell lines by qPCR. ^*^P < 0.01 compared to Sph. by two-tailed Student’s *t* test. (**D**) Level of NFATc3 protein was assessed in tumor spheres and their corresponding adherent monolayer cells derived from multiple OSCC cell lines by Western blot analysis.

Four isoforms of NFAT, NFATc1, NFATc2, NFATc3, and NFATc4, were identified [23]. To determine which of the NFAT isoforms are involved in OSCC progression, we first examined the expression level of four isoforms in two independent strains of normal human oral keratinocytes (NHOK-1 and NHOK-2), immortalized non-tumorigenic oral epithelial cell lines (NOKSI and HOK-16B), and OSCC cell lines (BapT, FaDu, SCC1, SCC4, SCC9/TNF, SCC15, SCC66, SCC105, SNU1066, UM1, UM2, UM6, UM17B, and YD38) by qPCR (Figure 1A). Among these four members, we found that NFATc3 was the dominant isoform and highly expressed in OSCC cells compared to the tested normal and immortalized cells. We noticed that the level of other NFAT isoforms (NFATc1, c2 and c4) was undetectable and/or negligible by both qPCR and Western blot analysis (Figure 1A and 1B). It is worthy to note that we observed gradual increase of NFATc3 expression in *in vitro* sequential, multistep oral carcinogenesis model, *i.e.,* NHOK→HOK-16BNHOK→BapT (Figure 1A and 1B). NHOK was immortalized by high-risk HPV-16 (HOK-16B cells), and HOK-16B was further transformed into oncogenic cells by the treatment of chemical carcinogen benzo(a)pyrene (BapT) [30].

Furthermore, we determined the level of NFATs in self-renewing CSCs (also known as tumor-initiating cells) that are responsible for tumor growth and aggressiveness [31]. CSC populations can be enriched in non-adherent tumor spheres cultured in ultra-low attachment plates that support the undifferentiated growth of self-renewing cells [32]. Therefore, abundance and the growth kinetics of non-adherent tumor spheres are indicative of self-renewing CSC content in a given culture of heterogeneous cancer cells. Tumor spheres derived from OSCC cells are CSC-enriched cell population as stemness transcription factors, NANOG, OCT4, KLF4, LIN28, and SOX2 were enriched in tumor spheres [19, 21]. To investigate an importance of NFATc3 in CSCs, we compared the levels of NFATc3 in tumor spheres and their corresponding adherent monolayer cells derived from multiple OSCC cell lines (Figure 1C and 1D). Similar to the result from Figure 1A, qPCR (Figure 1C) and western blot analysis (Figure 1D) revealed that NFATc3 is also the dominant isoform in tumor spheres, and its expression is enriched in tumor spheres compared to their corresponding adherent monolayer cells. Taken together, our findings indicate a stepwise elevation of NFATc3 expression during OSCC carcinogenesis and enrichment of NFATc3 in OSCC CSCs, suggesting an important role of NFATc3 in the progression of OSCC.

### Ectopic expression of NFATc3 converts non-tumorigenic immortalized oral epithelial cells to malignant phenotypes

**Figure 2:**
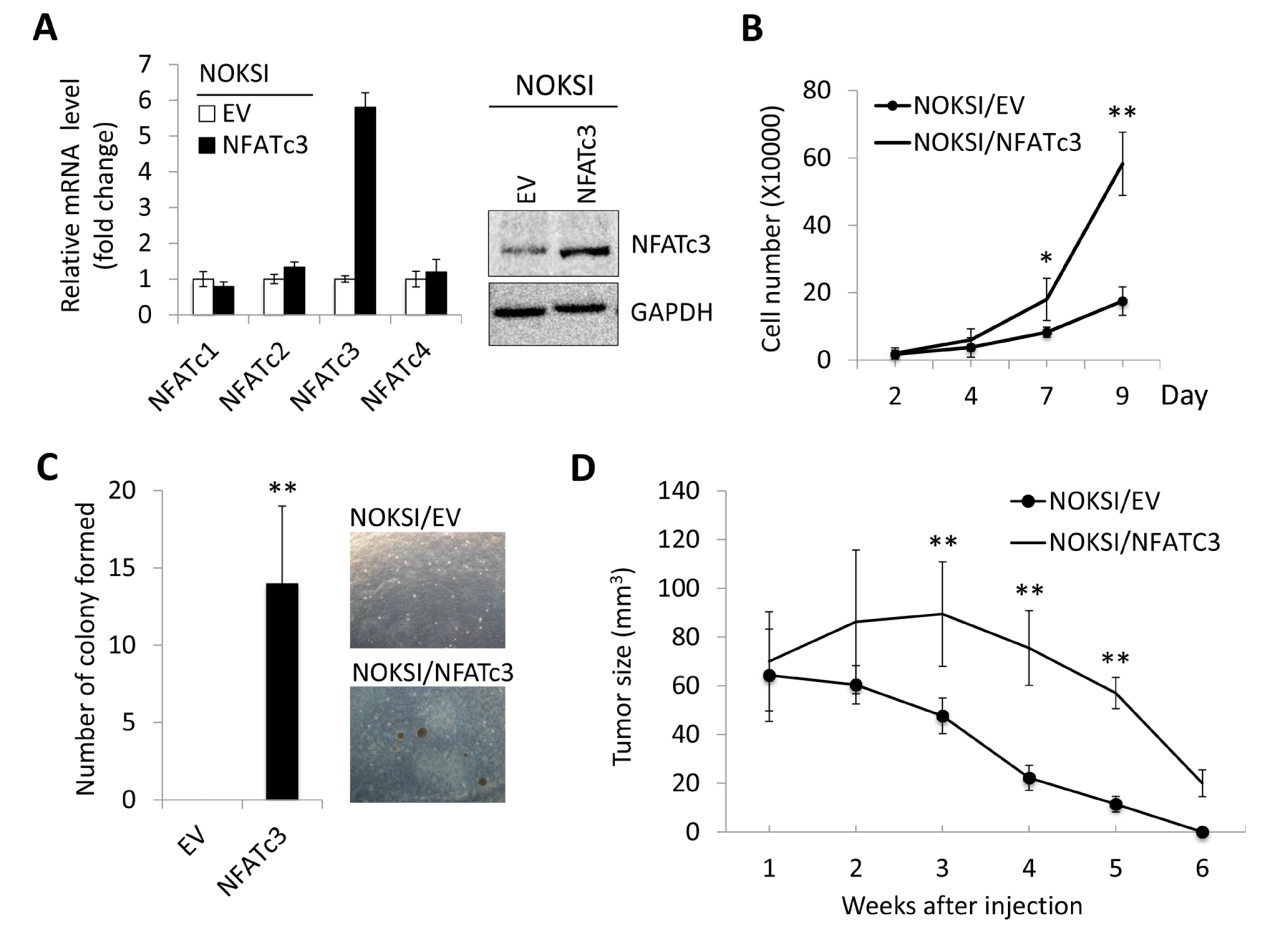
Ectopic expression of NFATc3 induces malignant cell growth in immortalized oral epithelial cells NFATc3 expression was forced in non-tumorigenic immortalized oral epithelial cells, NOKSI, by transfecting with vector expressing NFATc3 or empty vector (EV) as a control. (**A**) Ectopic expression of NFATc3 was confirmed by qPCR and Western blot analysis. (**B**) Effect of NFATc3 on cell proliferation was determined by cell counting. Data are means ± SD of triplicate experiments. ^*^P < 0.05 and ^**^P < 0.01 by two-tailed Student’s *t* test. (**C**) Effect of NFATc3 on anchorage independent growth ability was determined by soft agar assay. Ten thousand cells were plated in semi-solid agar, and colonies were counted for three weeks. The assay was performed in triplicate with 60-mm dishes. The photographs were taken at a magnification of 40X. (**D**) Effect of NFATc3 on *in vivo* tumorigenicity was determined by xenograft tumor assay. NOKSI/EV and NOKSI/NFATc3 were injected subcutaneously into 5 nude mice. Tumor sizes were measured for 6 weeks. ^**^P < 0.01.

Having established that increased NFATc3 is associated with OSCC progression, we investigated whether ectopic NFATc3 expression confers malignant cell growth traits on non-tumorigenic immortalized oral epithelial cells. As shown in Figure 2A, we overexpressed NFATc3 in spontaneously immortalized oral epithelial cells, NOKSI [33], using the vector expressing NFATc3 or empty vector (EV) as a control. We first examined the effect of NFATc3 on cell proliferation and found that NFATc3 overexpression led to robust increase in proliferation capacity *in vitro* (Figure 2B). NFATc3 conferred anchorage-independent growth ability to NOKSI cells (Figure 2C). As expected, the control NOKSI cells failed to show anchorage-independent growth ability. This ability has been linked to tumor cell aggressiveness *in vivo*, including tumorigenicity [34]. To further examine the effect of NFATc3 on tumor growth *in vivo*, we injected the cells into nude mice and observed tumor formation (Figure 2D). No tumors developed in mice receiving NOKSI/EV cells. However, NOKSI/NFATc3 cells began to form tumor nodules at 2 week after injection and reached their maximum size by 3 week. The nodules then regressed, leaving only necrotic tissue by 6 week. Our findings indicate that ectopic expression of NFATc3 led to the acquisition of malignant growth phenotype in the non-tumorigenic immortalized oral epithelial cell.

### NFATc3 is required to maintain CSC phenotype in OSCC

**Figure 3:**
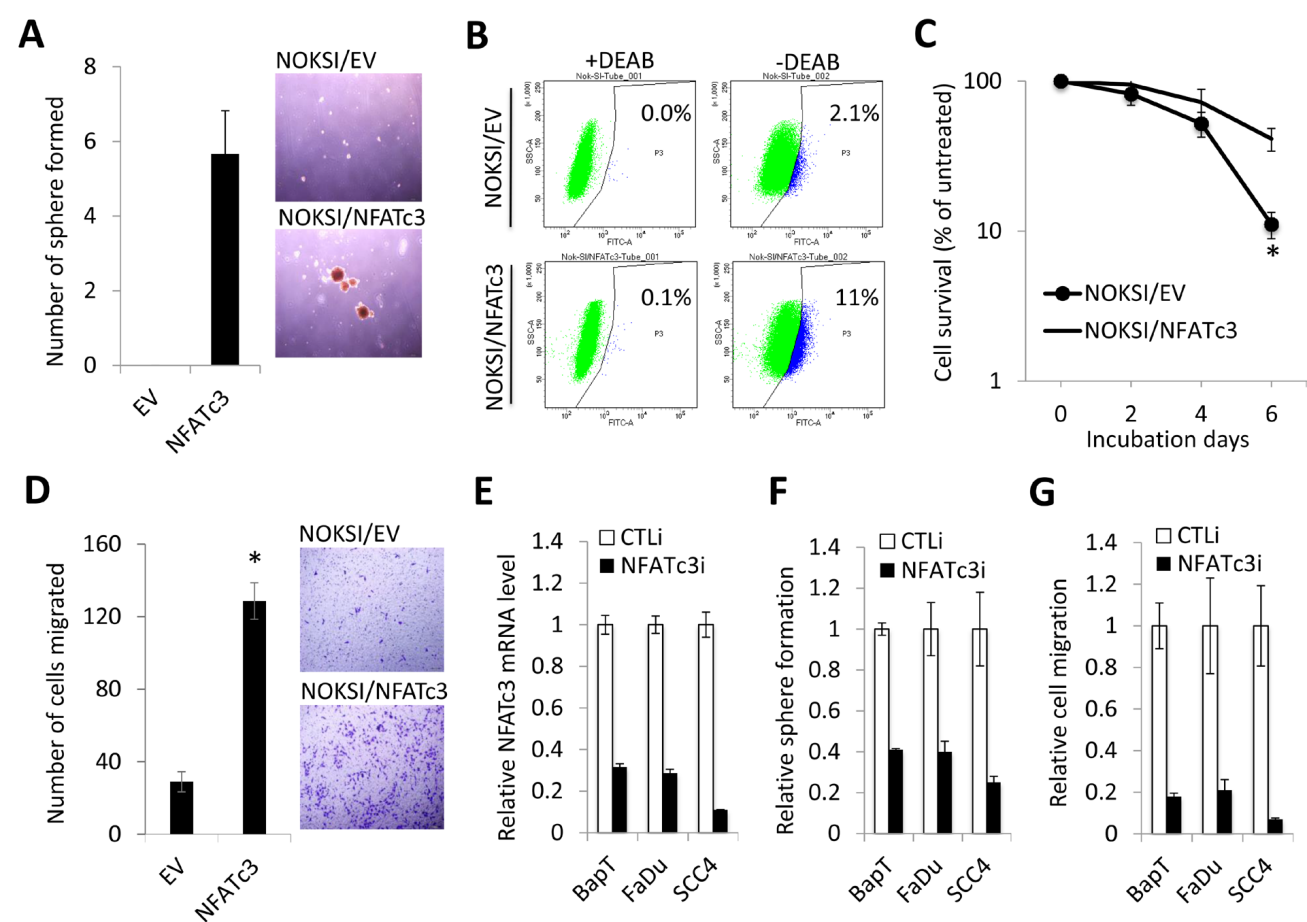
Increased NFATc3 is required to maintain CSC phenotype in OSCC (**A**) Effect of NFATc3 on self-renewal capacity of NOKSI was determined by tumor sphere formation assay. Representative image of tumor spheres formed by NOKSI/EV and NOKSI/NFATc3 are shown on the right. Bar indicates 100 μm. (**B**) Effect of NFATc3 on ALDH1 activity of NOKSI was determined by Aldefluor assay. Cells were labeled with Aldefluor combined with or without the ALDH1 inhibitor DEAB and analyzed by flow cytometry. The gate for ALDH1^HIGH^ cells is determined in relation to the DEAB control (+DEAB) and shows the brightly fluorescent ALDH1 population versus the side scatter, a population that is absent/decreased in the presence of DEAB. The number shown in each panel reflects the percentage of ALDH1^HIGH^ cells in each cell type. (**C**) Effect of NFATc3 on chemoresistance of NOKSI was determined by MTT assay. Cells were treated with 40 µM of cisplatin for 2, 4, and 6 days, and their viability was determined. Data are expressed as the mean ± SD of triplicate. ^*^P < 0.01 **(D**) Effect of NFATc3 on migration ability of NOKSI was determined by transwell migration assay. ^*^P < 0.01. Representative images of transwell migration assay are shown on the right. (**E**) Endogenous NFATc3 was knocked down in multiple OSCC cell lines using siRNA against NFATc3 (NFATc3i). The cells transfected with control siRNA (CTLi) were included for comparison. Knockdown of NFATc3 was confirmed by qPCR. (**F**) The effect of NFATc3 knockdown on self-renewal capacity was determined by tumor sphere formation assay. (**G**) The effect of NFATc3 knockdown on migration ability was determined by transwell migration assay.

Next, we investigated the effect of NFATc3 on CSC phenotype in NOKSI. Ectopic NFATc3 expression resulted in robust induction in tumor sphere formation, indicating the acquisition of self-renewal capacity by NFATc3 (Figure 3A). Activity of aldehyde dehydrogenase 1 (ALDH1) has been widely used as markers for isolating CSCs. ALDH1^HIGH^ cancer cells displayed higher CSC properties compared to ALDH1^low^ cells [35–37]. Thus, to test whether ectopic NFATc3 expression increases ALDH1^HIGH^ CSC population, we sorted ALDH1^HIGH^ and ALDH1^low^ cells from NOKSI/EV and NOKSI/NFATc3 cells by performing flow cytometry analysis. The assay revealed a significant increase in ALDH1^HIGH^ cell population in NOKSI/NFATc3 compared to their control NOKSI/EV (11.0% vs. 2.1%; Figure 3B). Another important characteristic of CSCs is their resistance to anticancer drugs [31]. Cisplatin is the most common anticancer drug for head and neck cancer. The NOKSI/NFATc3 cells displayed increased resistance to cisplatin compared to their control NOKSI/EV cells (Figure 3C). We also measured the level of NFATc3 expression from parental SCC4 cells and cisplatin-resistant SCC4 cells that were isolated from SCC4 treated with 25 μM cisplatin for 2 days. Level of NFATc3 was highly expressed in cisplatin-resistant SCC4 cells compared to their parental control (Supplementary Figure 1). Because metastatic potential is well-known property of CSCs [31], we also examined the effect of NFATc3 on migration ability of NOKSI. As demonstrated by transwell migration (Figure 3D), NFATc3 increased migration ability of the cells. Our findings indicate that ectopic NFATc3 expression is sufficient to confer CSC phenotype on the immortalized oral epithelial cells.

Conversely, we knocked down NFATc3 in multiple OSCC cell lines, BapT, FaDu, and SCC4, by using siRNA (Figure 3E). Knockdown of NFATc3 reduced tumor sphere formation in OSCC cells (Figure 3F). As demonstrated by transwell migration (Figure 3G), knockdown of NFATc3 suppressed migration ability of OSCC cells. To extend these observations, we also determined whether chemical inhibition of NFAT suppresses CSC phenotype. NFAT is activated by a protein phosphatase complex of calmodulin and calcineurin. Cyclosporine A (CsA) is a potent calcineurin inhibitor, thus an indirect inhibitor of NFAT [38]. We treated OSCC cells with CsA, and subsequently performed the assays for CSC properties. The NFAT inhibitor significantly inhibited self-renewal (Supplementary Figure 2A) and migration (Supplementary Figure 2B) of OSCC cell lines, SCC4 and FaDu. The inhibitory effect of CsA on self-renewal and migration capacity of SCC4 was also used in our previous publication as Supplementary Figure 3 [21]. Overall, our data clearly indicate that NFATc3 is required for maintenance of CSC phenotype in OSCC.

### NFATc3 upregulates OCT4 by activating its promoter activity

**Figure 4:**
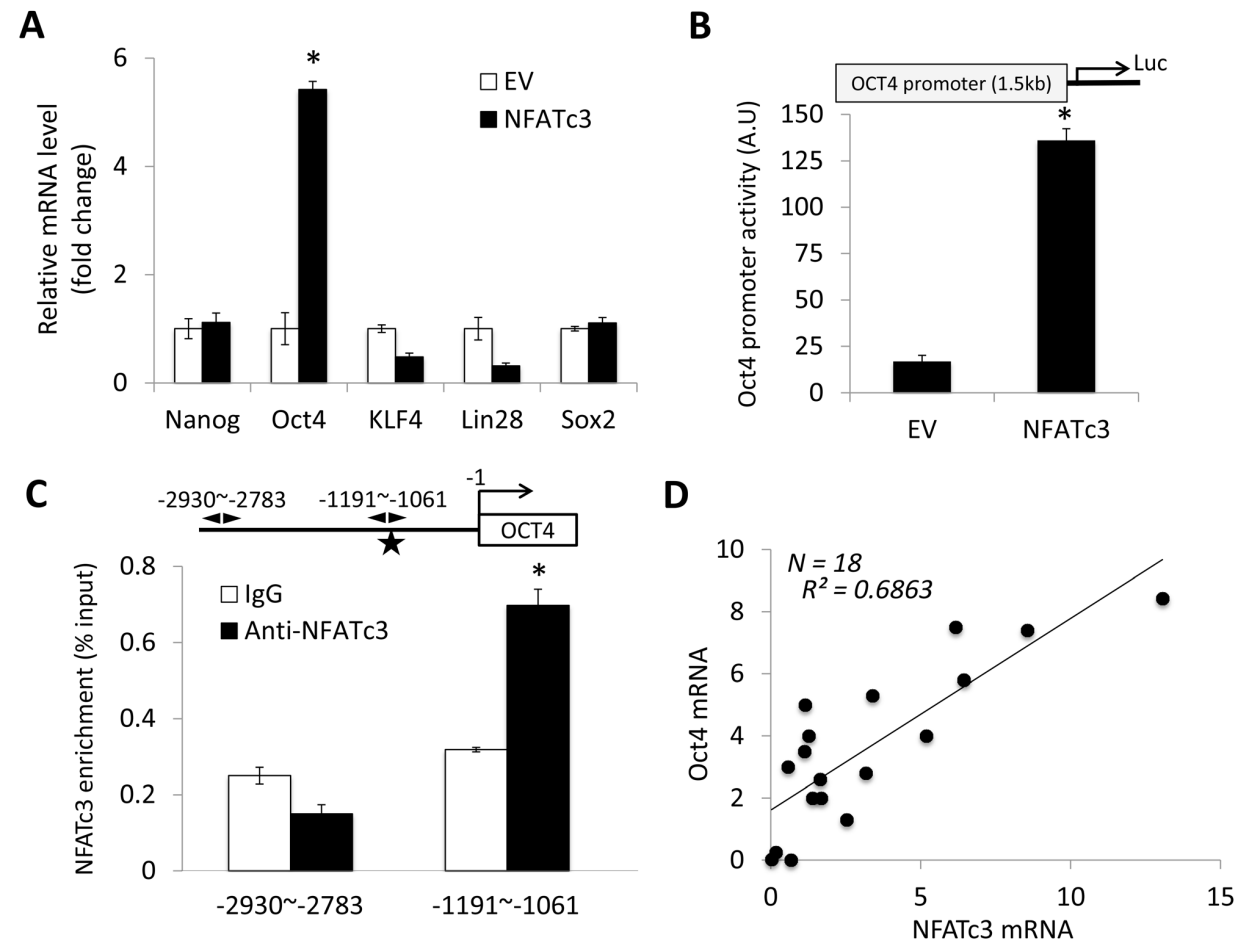
NFATc3 increases OCT4 expression by activating OCT4 promoter (**A**) Effect of NFATc3 on pluripotent transcription factors (NANOG, OCT4, KLF4, LIN28, and SOX2) expression was determined by qPCR. Their levels in NOKSI/NFATc3 were plotted as fold change against those in NOKSI/EV. ^*^P < 0.001. (**B**) Effect of NFATc3 on OCT4 promoter activity was determined by luciferase promoter assay. Cells were transfected with pGL3-Basic (promoter-less) or pGL3 vectors containing the 1.5-kb upstream (-1545∼ -24) of Oct4. ^*^P < 0.001. (**C**) Sequence analysis reveals a consensus NFAT binding site (5′-GGAAA-3′) at -1088 ∼ -1084 indicated by star (upper diagram). OSCC cells were lysed and performed a ChIP assay. The fragment (-1191∼ -1061) containing the NFAT binding site was enriched with NFATc3, and the fragment (-2930∼ -2783) was amplified as a control. ^*^P < 0.01. (**D**) Correlation analysis of NFATc3 and OCT4 mRNA was determined based on their expression levels in 18 human SCC cell lines by qPCR.

Pluripotent transcription factors, NANOG, OCT4, KLF4, LIN28 and SOX2, play the major role in regulating stemness properties, including self-renewal [39]. Indeed, we demonstrated their overexpression in self-renewing OSCC tumor spheres compared to their corresponding adherent cells [19, 21]. Thus, we sought to determine whether there was a functional link between NFATc3 and pluripotent transcription factors in regulating OSCC CSCs. We examined the effect of NFATc3 on their expression and observed that NFATc3 markedly increased OCT4 transcription (Figure 4A). To investigate the effect of NFATc3 on the promoter activity of OCT4, we performed luciferase reporter assay using the OCT4 promoter construct containing a 1.5-kb locus upstream (-1545∼ -24) from the transcription start site of OCT4 (Figure 4B). The activity of OCT4 promoter from NOKSI/NFATc3 was significantly greater than that from the control NOKSI/EV (Figure 4B). Our sequencing analysis identified a potential NFAT binding site (5′-GGAAA-3′ at -1088 ∼ -1084; Figure 4C) [40]. Thus, to further confirm the direct involvement of NFATc3 in the transcriptional activation of OCT4, we performed ChIP assay and demonstrated that a -1,191 ∼ -1,061 bp region of the OCT4 promoter was occupied by NFATc3 protein, indicating that NFATc3 physically associates with the OCT4 promoter *in vivo* (Figure 4C). Additionally, we demonstrated that NFATc3 transcription levels were positively correlated with the OCT4 transcription levels in 18 independent human SCC cell lines (Figure 4D). Similarly, the expression levels of NFATc3 protein (Figure 1B) were also positively associated with those of OCT4 protein (Supplementary Figure 3). Together, our findings indicate that OCT4 is a direct transcriptional target of NFATc3.

### OCT4 is required for NFATc3-induced CSC phenotype

**Figure 5:**
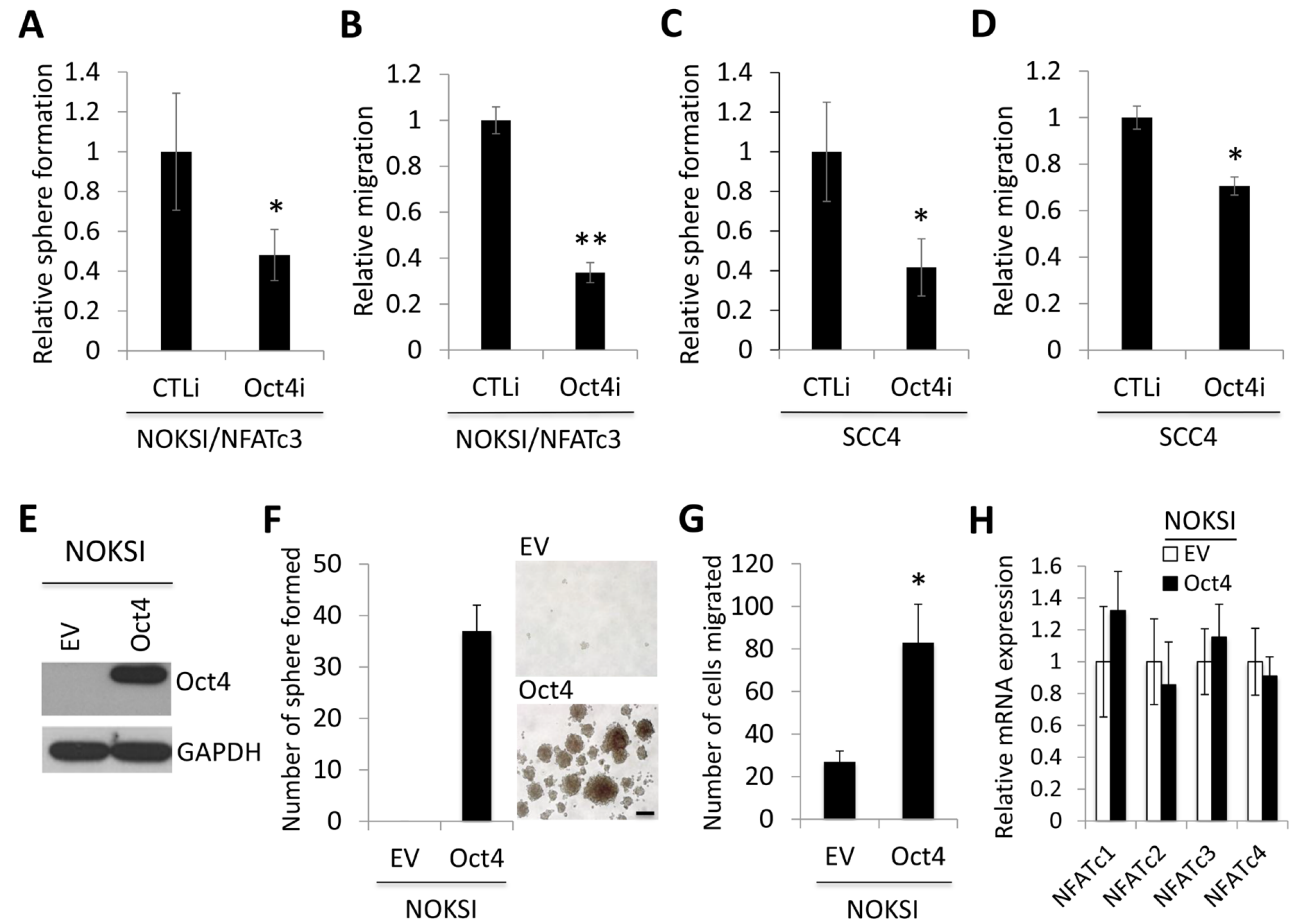
OCT4 is required for NFATc3-induced CSC phenotype (**A**) The effect of OCT4 knockdown on self-renewal capacity of NOKSI/NFATc3 was determined by tumor sphere formation assay. OCT4 was knocked down in NOKSI/NFATc3 using siRNA against OCT4 (Oct4i). The cells transfected with control siRNA (CTLi) were included for comparison. ^*^P < 0.05. (**B**) The effect of OCT4 knockdown on migration ability in NOKSI/NFATc3 was determined by transwell migration assay. ^**^P < 0.01. (**C**) The effect of OCT4 knockdown on self-renewal capacity of SCC4 was determined by tumor sphere formation assay. (**D**) The effect of OCT4 knockdown on self-renewal capacity of SCC4 was determined by transwell migration assay. (**E**) OCT4 expression was forced in non-tumorigenic immortalized oral epithelial cells, NOKSI, by vector expressing recombinant Myc-DDK-tagged OCT4, and its ectopic expression was confirmed by Western blot analysis using anti-DDK antibody. (**F**) Effect of ectopic OCT4 expression on self-renewal capacity of NOKSI was determined by tumor sphere formation assay. Representative images of tumor spheres formed by NOKSI/EV and NOKSI/Oct4 are shown on the right. (**G**) Effect of ectopic OCT4 expression on migration ability of NOKSI was determined by transwell migration assay. (**H**) Effect of ectopic OCT4 expression on the expression of NFAT isoforms (NFATc1-c4) in NOKSI was determined by qPCR. Their levels in NOKSI/Oct4 were plotted as fold induction against those in NOKSI/EV.

To evaluate the functional role of OCT4 in NFATc3-induced CSC phenotype, we knocked down OCT4 using siRNA in NOKSI/NFATc3. Knockdown of OCT4 showed significant suppressive effect on self-renewal (Figure 5A) and migration (Figure 5B) in NOKSI/NFATc3 cells. We also demonstrated that knockdown of OCT4 resulted in significant suppression of tumor sphere formation (Figure 5C) and migration ability (Figure 5D) in OSCC cells. Conversely, we overexpressed OCT4 in NOKSI (Figure 5E) and determined its effects on CSC phenotype. Ectopic OCT4 expression resulted in robust induction in tumor sphere formation, indicating the acquisition of self-renewal capacity by OCT4 (Figure 5F). OCT4 also increased migration ability of the cells (Figure 5G). Furthermore, we examined whether OCT4 affected NFATc3, and found no significant changes in NFATc3 expression by OCT4 (Figure 5H), indicating that NFATc3 lies upstream of OCT4. Collectively, our data indicate that NFATc3 promotes CSC phenotype by upregulating OCT4, suggesting a novel CSC regulatory mechanism by the NFATc3-OCT4 axis.

### Elevated expression of NFATc3 is associated with OSCC carcinogenesis *in vivo*

**Figure 6:**
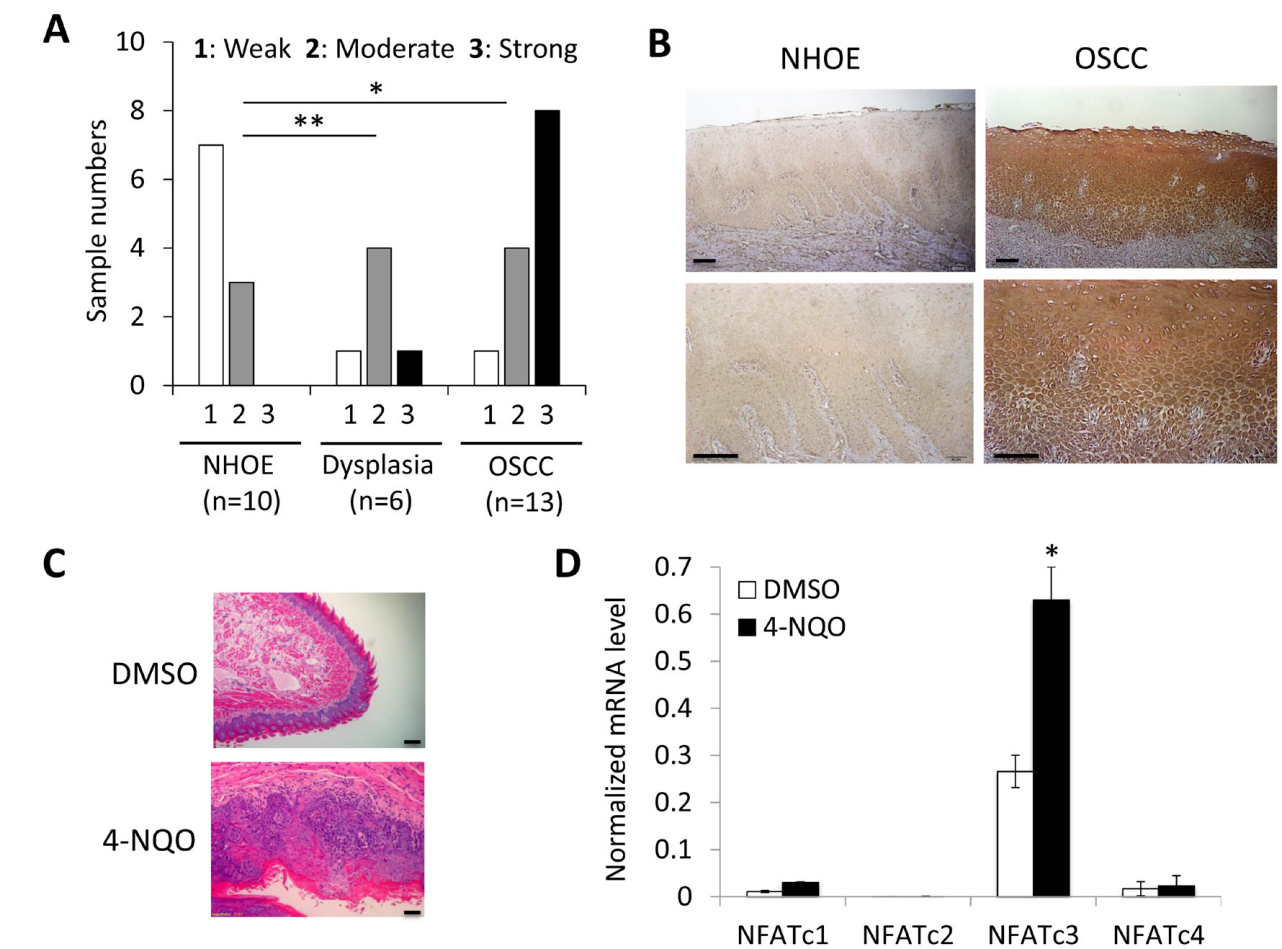
Elevated expression of NFATc3 during OSCC carcinogenesis *in vivo* (**A**) *In vivo* NFATc3 expression was determined in normal human oral epithelia (NHOE), oral dysplasia and OSCC tissues by immunohistochemical (IHC) staining. ^*^P < 0.01 and ^**^P < 0.05. (**B**) Representative examples of NFATc3 IHC staining in NHOE and OSCC tissues *in vivo*. Bar indicates 100 μm. (**C**) Morphological change of tongues from mice treated without or with 4-NQO (30 µg/ml) for 4 months to induce oral cancer formation. Bar indicates 50 μm. (**D**) Level of NFAT isoforms (NFATc1-c4) was determined in tongues from mice exposed to DMSO or 4-NQO by qPCR. The levels of 4 NFAT isoforms were normalized to GAPDH. qPCR was performed with total RNAs isolated from tongue tissues.

To further confirm the role of NFATc3 on OSCC carcinogenesis *in vivo*, immunohistochemical (IHC) staining for NFATc3 was performed using normal human oral epithelia (NHOE), oral dysplasia, and OSCC tissues. The results of *in vivo* NFATc3 staining are summarized in Figure 6A, and a typical NFATc3 staining is shown in Figure 6B. In 10 NHOE, weak NFATc3 staining was detected in 7 cases (70%), and moderate staining detected in 3 cases (30%). Of 6 dysplastic tissues, weak staining was detected in 1 case (16.7%), moderate staining detected in 4 cases (66.7%), and strong staining detected in 1 case (16.7%). In 13 OSCC samples, 4 cases (30.8%) demonstrated moderate staining and 8 cases (61.5%) with strong staining. Mean IHC scores for NFATc3 in NHOE, dysplasia, and OSCC were 1.30, 2.0, and 2.54, respectively, showing statistical significant difference (Figure 6A). Together with our *in vitro* findings (Figure 1), these data confirmed a stepwise elevation of NFATc3 expression in OSCC progression.

Lastly, to confirm the importance of elevated NFATc3 during oral carcinogenesis, we utilized a carcinogen-induced tongue cancer mouse model which was demonstrated in our publication [41]. In the model, chronic exposure of mice to 4-nitroquinoline-1-oxide (4-NQO) led to rampant oral tumor formation in the tongue, while tongue from mice exposed to DMSO (vehicle) exhibited similar histology with normal squamous epithelium (Figure 6C). Consistent with our findings from human cancer, NFATc3 is also the dominant isoform among NFAT isoforms (NFATc1-c4) and significantly increased in the tumor bearing tongue compared to normal tongue, suggesting that increased NFATc3 is associated with chemical-induced oral carcinogenesis (Figure 6D).

## DISCUSSION

Our findings reveal for the first time that NFATc3-OCT4 signaling is a novel molecular axis for cancer stemness of OSCC. Among NFAT isoforms (NFATc1-c4), NFATc3 is the dominant isoform in oral epithelial cells, and its expression is elevated in a stepwise manner during OSCC carcinogenesis. NFATc3 expression is further enriched in self-renewing CSC populations in OSCC. Ectopic NFATc3 expression increases CSC population and property in immortalized oral epithelial cells. Conversely, inhibition of NFATc3 suppresses CSC phenotype in OSCC. Moreover, our study reveals that NFATc3 is a novel transcription regulator for OCT4. We provide the evidence that NFATc3 enhances CSC phenotype by upregulating OCT4.

NFATs play an important role in tumorigenesis by regulating downstream targets involved in cancer development [22]. For instance, NFATc1 induced malignant growth phenotype in pancreatic cancer cells by upregulating MYC [42] and promoted metastasis of mammalian cancer cells *via* MMP-2 upregulation [43–45]. NFATc2 was overexpressed in multiple cancer types [44, 45], and its depletion suppressed migration/invasion of cancer cells [44]. NFATc3 was highly expressed in gastric cancer tissues [46] and promoted tumor progression and aggressiveness [47, 48]. However, other studies also demonstrated tumor suppressive activity of NFATc2 and NFATc3 [24, 25], suggesting NFAT isoforms play different roles in human cancers in different cellular context. Thus, it remians to be clarified 1) which NFAT isoform is dominant in OSCC and 2) its role in OSCC carcinongenesis. Our results showed that NFATc3 is most abundant isoform and highly expressed in OSCC compared to precancerous and normal oral epithelial cells and tissues. To our knowledge, our finding is the first report showing a stepwise elevation of NFATc3 during the progression of OSCC. Moreover, ectopic expression of NFATc3 resulted in the acquisition of malignant growth phenotype in the non-tumorigenic immortalized oral epithelial cells, indicating the function of NFATc3 in cell transformation and cancer progression. Thus, we hypothesize that NFATc3 is a novel oncogenic driver in oral/oropharyngeal tumorigenesis.

By functional and phenotypic analysis, we demonstrate that NFATc3 is an important regulator of CSC phenotype in OSCC. NFATc3 is highly expressed in CSC-enriched self-renewing OSCC populations, *i.e.,* tumor spheres. Furthermore, NFATc3 endowed immortalized oral epithelial cells with self-renewal and concomitantly increased ALDH1^HIGH^ CSC population. ALDH1 has been found to be a marker for stem cells in different types of cancer, including OSCC [35–37]. ALDH1^HIGH^ cancer cells displayed higher self-renewal, migration, and tumorigenic potential than ALDH1^low^ cells [49–51]. Suppression of NFATc3 inhibited self-renewal capacity in multiple OSCC cell lines. Our data indicate that NFATc3 is required to maintain self-renewal capacity. Since self-renewal represents the driving force of tumor progression and metastasis, our findings are of paramount important for the development of more effective cancer therapies. NFATc3 also regulated important CSC properties, such as migration and chemoresistance. Our finding is consistent with previous reports showing the importance of NFATc3 in cell migration [52, 53]. However, underlying mechanism by which NFATc3 regulates OSCC migration has not been understood. Therefore, effects of NFATc3 on epithelial-to-mesenchymal transition (EMT) and metastasis-related gene expression should be warranted to investigate [54]. We demonstrated that ectopic NFATc3 expression increased cisplatin resistance. Similarly, NFATc2 overexpression increased cisplatin resistance in a lung cancer cell line [29]. Moreover, REDD1 (regulated in development and DNA damage response-1) was known to a target of NFATc3, and its inhibition sensitized human cancer cells to paclitaxel [55, 56]. Thus, the role of REDD1 in NFATc3-induced chemoresistance should be warranted to investigate. We conclude that NFATc3 is required to maintain CSC phenotype. Therefore, NFATc3 could be an effective therapeutic target for OSCC.

Our study demonstrates that NFATc3 is a transcriptional activator of OCT4, the key transcription factor that is required for the stemness properties of embryonic stem cells [57]. Importantly, OCT4 is highly expressed in OSCC cell lines and tissues [7, 58]. These findings suggest that OCT4 may play an oncogenic role in tumorigenesis. Moreover, it has been demonstrated that OCT4 is involved in the maintenance of CSCs, including OSCC CSCs [7, 59, 60]. For instance, overexpression of OCT4 converted differentiated OSCC cells into stem-like OSCC cells [60], and the abrogation of OCT4 in OSCC cells suppressed self-renewal capacity and tumor growth *in vivo* [59]. OCT4 regulates the transcription of many genes in CSCs; however, how OCT4 expression is regulated by upstream signals remains obscure [61]. Here, we observed positive correlation between NFATc3 and OCT4 transcription levels. We also demonstrated that NFATc3 can bind to the promoter region of OCT4 at -1,191 ∼ -1,061 bp where the consensus NFAT binding site was identified, thereby activating OCT4 transcription. In addition, we also found that NFATc3 expression resulted in robust induction in ZEB1 and ZEB2 (data not shown). Their expressions are significantly increased in head and neck CSCs compared to non-CSCs [62]. Knockdown of ZEB1 and ZEB2 in head and neck cancer cells decreased their CSC properties such as self-renewal capacity, the expression of stemness markers, and drug resistance. Moreover, their suppression inhibited *in vivo* tumor growth and the rate of metastasis to distant site [62]. Conversely, co-overexpression of ZEB1 and ZEB2 enhanced sphere-forming ability of head and neck cancer cells [62]. Our observations so far support the hypothesis that NFATc3 promotes cancer stemness by upregulating CSC factors, including OCT4.

Activation of NFAT requires increased intracellular Ca^2+^ concentration that is mainly mediated by ORAI1 Ca^2+^ channels in non-excitable cells [63]. ORAI1-mediated Ca^2+^ influx leads to the dephosphorylation of NFAT by activation of a protein phosphatase complex of calmodulin and calcineurin, resulting in its translocation from the cytoplasm to the nucleus [64, 65]. Although underlying mechanism of increased NFATc3 in CSCs is obscure, our recent study suggested the role of ORAI1 in NFATc3 activation [21]. ORAI1 is overexpressed in different types of cancer including OSCC. We recently reported that ORAI1 is enriched in CSCs, and its suppression abolished CSC phenotype in OSCC. Overexpression of ORAI1 increased intracellular Ca^2+^ level and CSC phenotype in oral epithelial cells. Moreover, NFATc3 expression is upregulated by ORAI1 and downregulated by inhibition of ORAI1. Since OCT4 is a novel target of NFATc3, the major downstream effector molecule of ORAI1 [21], we speculate a possible role of ORAI1-NFATc3-OCT4 axis in CSC regulation.

In conclusion, NFATc3 is a novel molecular regulator of stemness of OSCC. NFATc3 enhances CSC phenotype through OCT4 signaling. Thus, the NFATc3-OCT4 axis could be an important therapeutic target in OSCC. Since chemical inhibitor readily inhibits NFAT signaling, targeting NFATc3 may be a plausible therapeutic modality against cancer.

## MATERIALS AND METHODS

### Cell culture and reagents

Primary normal human oral keratinocytes (NHOK) were prepared from oral mucosa and cultured in Keratinocyte Growth Medium (KGM, Lonza) as described previously [66]. Two non-tumorigenic immortalized oral epithelial cell lines, NOKSI [33] and HOK-16B [66], were also cultured in KGM. Fourteen human OSCC cell lines (BapT, FaDu, SCC1, SCC4, SCC9/TNF, SCC15, SCC66, SCC105, SNU1066, UM1, UM2, UM6, UM17B, and YD38) were cultured in DMEM/Ham’s F12 (Invitrogen) supplemented with 10% FBS (Gemini Bioproducts) and 0.4 µg/ml hydrocortisone (Sigma-Aldrich). Antagonist of NFAT signaling, cyclosporine A (CsA), was purchased from Sigma-Aldrich.

### Quantitative real-time PCR (qPCR)

cDNA was synthesized from 5 μg of total RNA using SuperScript first-strand synthesis system (Invitrogen). We used 1 μl cDNA for qPCR amplification using SYBR Green I Master mix (Roche) and LightCycler 480 II (Roche). The primer sequences for NFATc1-c4 were obtained from the Universal Probe Library (Roche), and the sequences can be available upon request. Second derivative Cq value determination method was used to compare fold-differences according to the manufacturer’s instructions.

### Western blotting

Western blotting was performed as described previously [67]. We used the following primary antibodies for our study: NFATc1 (8032S; Cell Signaling Tech), NFATc2 (sc-7296; Santa Cruz Biotech), NFATc3 (sc-8405; Santa Cruz Biotech), NFATc4 (sc-13036; Santa Cruz Biotech), C-MYC (sc-40; Santa Cruz Biotech), α-tubulin (T9026; Sigma), GAPDH (FL-335; Santa Cruz Biotech), and DDK (TA50011-100; OriGene).

### Tumor sphere formation assay

Three thousand cells were grown in 3 ml of serum-free DMEM/F12 media supplemented with 1:50 B27 (Invitrogen), 20 ng/mL EGF, 20 ng/mL, 10μg/mL insulin, penicillin, streptomycin, and amphotericin B in Ultra-Low Attachment 6-well Plates (Corning) for 6-10 days [67].

### Ectopic expression of NFATc3 or Oct4

The mammalian pREP Vector containing NFATc3 was purchased from Addgene (#11790). The mammalian pCMV6-Entry Vector encoding OCT4 was purchased from OriGene (RC211998). Detailed methods of transfection and selection can be found in our previous publications [68].

### Small interfering RNA (siRNA) transfection

Specific siRNA (targeting NFATc3 or Oct4) and control siRNA were purchased (Santa Cruz Biotech) and introduced into cells using Lipofectamine RNAiMAX (Invitrogen). Cells (2 × 10^5^) were plated in 60-mm dishes and transfected with 15 μg siRNA. The cultures were harvested after two days post-transfection for expression and functional analyses.

### Anchorage-independent growth

To determine colony-forming efficiency in semi-solid medium, 1 × 10^4^ cells were plated in culture medium containing 0.3% agarose over a base layer of serum-free medium containing 0.5% agarose. Three weeks after incubation, colonies were counted. The experiment was performed in triplicates with 60-mm dishes.

### *In vivo* xenograft tumor assay

Five million cells were subcutaneously injected into the flank of immunocompromised mice (strain *nu/nu*, Charles River Laboratories). The animal study was performed according to the protocol approved by UCLA Animal Research Committee. The kinetics of tumor growth was determined by measuring the volume in three perpendicular axes of the nodules using micro-scaled calipers.

### ALDH1 assay

Using Aldehyde Dehydrogenase-Based Cell Detction Kit (STEMCELL), ALDH enzymatic activity was determined. Total of 1 × 10^6^ cells were re-suspended in the ALDEFLUOR Assay Buffer in the volume of 1 ml. Fluorescent nontoxic ALDEFLUOR Reagent BODIPY^™^ (1.25 µl) was added as a substrate to measure ALDH enzymatic activity in intact cells. Immediately after adding the substrate reagent, 0.5ml of the cell suspension was transferred into the control tube which contains specific inhibitor for ALDH, diethylaminobenzaldehyde (DEAB) for calculating background fluorescence. Then, cells were incubated at 35° C for 30 minutes and fluorescence data acquisition was made by using a BD FACScan flow cytometer (BD Biosciences).

### Migration assay

Cell migration was measured using transwell chambers with polycarbonate membranes (Corning) according to the method as described in manufacture protocol as described in our previous publication [67].

### Chemo-sensitivity assay

Chemosensitivity of cells was determined by measuring cell viability using the tetrazolium salt (MTT) cell proliferation assay kit (ATCC). The cells were plated at 2 × 10^3^ cells per well into 96-well plate and incubated in culture medium containing 50 µM cisplatin (Sigma-Aldrich) for 2, 4, and 6 days. Absorbance at 570 nm was determined using a microplate reader.

### OCT4 promoter constructs and luciferase assay

To generate human OCT4 promoter reporter construct, a 1.5 kb upstream region of human OCT4 sequence (-1545∼ -24) was retrieved by digesting hOCT4-Luc (a gift from Shinya Yamanaka, Addgene plasmid #17221) with *Sac*I and *Bgl*II and subcloned into pGL3-basic vector (Promega). Transfection and luciferase assay were carried out as described in previous publications [68].

### Chromatin immunoprecipitation (ChIP)

ChIP was performed using a ChIP assay kit (Upstate) according to the manufacturer’s protocol. Immune complexes were obtained using 5 µg of NFATc3 antibody (sc-1152; Santa Cruz Biotech). Then, genomic DNA was isolated from the complexes and subjected to qPCR using the OCT4 promoter primers (forward for -1191∼ -1061, 5′-GGGAGCAAGGAACCTGATGTG-3′; reverse for -1191∼ -1061, 5′-TTTGGACTGACTGGG CCTC-3′; forward for -2930∼ -2783, 5′-TGCCTCAACCTCCCATCAG -3′; reverse for -2930∼ -2783, 5′-AGAGGGACGCAGACAAGG-3′) as described previously [19].

### Immunohistochemistry

Tissue specimens that were previously collected for diagnostic purposes were obtained from the Oral Pathology Diagnostic Laboratory at the UCLA School of Dentistry. All tissue specimens were collected and processed according to the guidelines of the University of California at Los Angeles Institutional Review Board. Immunohistochemical staining was performed as described previously [69]. The optimal concentration (1:100) of NFATc3 antibody (sc-8405; Santa Cruz Biotech) was first established using serially diluted primary antibody along with IgG as a negative control. The level of NFATc3staining pattern was scored into three subgroups: (1) weak; (2) moderate; and (3) strong.

### Mouse models

To induce oral tumors, C57BL/6 mice (Jackson Laboratory) were exposed to 4-NQO (Sigma-Aldrich, St. Louis, MO) diluted in drinking water to the final concentration of 30 µg/ml for 16 weeks, followed by six weeks of normal drinking water, as described in our previous study [41]. At the end of experiment, tongues were harvested for histological examination and RNA isolation. All procedures involving the use of mice will be in accordance with the National Institutes of Health guidelines and are approved by the UCLA Animal Research Committee.

## SUPPLEMENTARY MATERIALS FIGURES


