# Supplementary material for: NFATc3 plays an oncogenic role in oral/oropharyngeal squamous cell carcinomas by promoting cancer stemness via expression of OCT4
Source: Oncotarget. 2019 Mar 19;10(23):2306–19. doi: 10.18632/oncotarget.26774 (PMC6481346; doi:10.18632/oncotarget.26774)
Supplement: Supplementary file 1 [file oncotarget-10-2306-s001.pdf]

NFATc3 plays an oncogenic role in oral/oropharyngeal squamous cell carcinomas by promoting cancer stemness via expression of OCT4

SUPPLEMENTARY MATERIALS

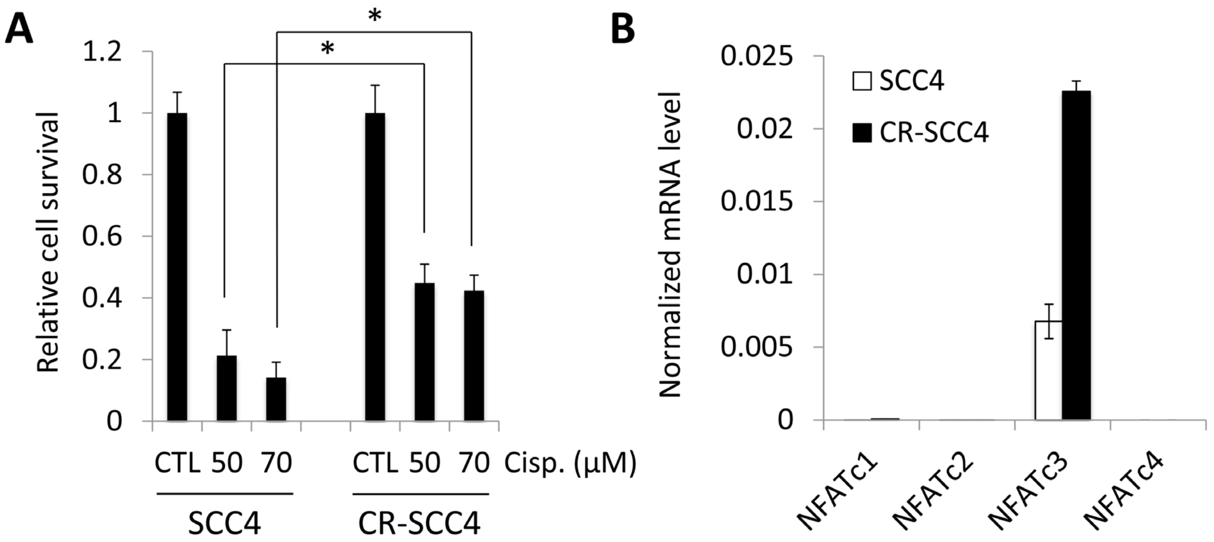

Supplementary Figure 1: NFATc3 expression is increased in drug-resistant OSCC cells. Cisplatin-resistant SCC4 (CR-SCC4) cells were isolated from SCC4 treated with 25 μM cisplatin for 2 days. (**A**) Cisplatin-sensitivity of SCC4 and CR-SCC4 was determined by MTT assay. Cells were treated with 50 μM or 70 μM of cisplatin for 48 hours, and their viability was determined. Data are expressed as the mean ± SD of triplicate. $^{*}P < 0.05$ (**B**) Level of NFAT isoforms (NFATc1-c4) was determined in parental SCC4 and CR-SCC4 by qPCR. Levels of NFAT isoforms were normalized to GAPDH.

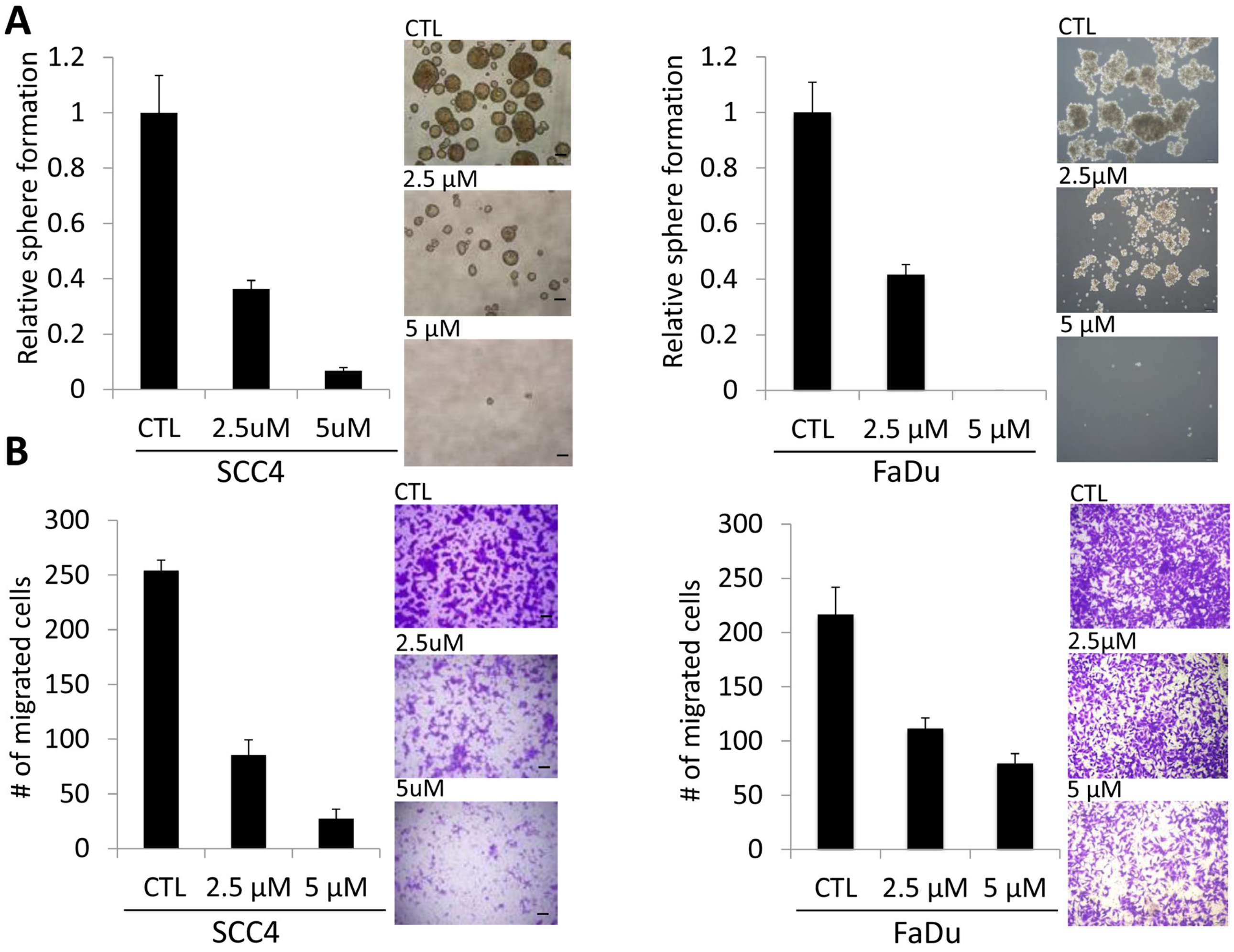

Supplementary Figure 2: NFAT antagonist suppresses CSC properties in OSCC. (**A**) Effect of NFAT antagonist, cyclosporine A (CsA), on self-renewal capacity of OSCC cells (SCC4 and FaDu) was determined by tumor sphere formation assay. Representative images of tumor spheres formed are shown on the right. (**B**) Effect of the NFAT antagonist on migration ability in OSCC cells was determined by transwell migration assay. Representative images of transwell migration assay are shown on the right.

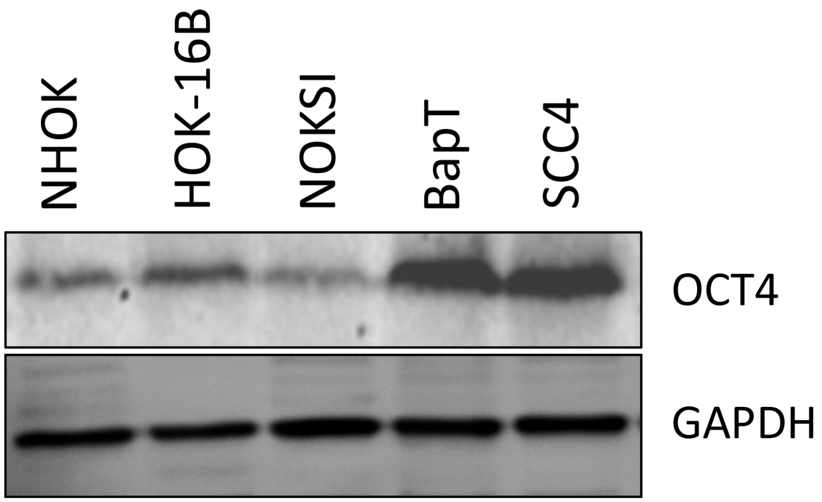

Supplementary Figure 3: OCT4 protein expression in various human oral epithelial cells. Level of OCT4 protein was determined in normal (NHOK), precancerous (HOK-16B and NOKSI) and OSCC cells (BapT and SCC4) by Western blot analysis. GAPDH was used as loading control.
